# Propofol Pharmacokinetics and Estimation of Fetal Propofol Exposure during Mid-Gestational Fetal Surgery: A Maternal-Fetal Sheep Model

**DOI:** 10.1371/journal.pone.0146563

**Published:** 2016-01-11

**Authors:** Pornswan Ngamprasertwong, Min Dong, Jing Niu, Raja Venkatasubramanian, Alexander A. Vinks, Senthilkumar Sadhasivam

**Affiliations:** 1 Department of Anesthesiology, Cincinnati Children’s Hospital Medical Center, Cincinnati, OH, United States of America; 2 Division of Clinical Pharmacology, Department of Pediatrics, Cincinnati Children’s Hospital Medical Center, Cincinnati, OH, United States of America; 3 Department of Anesthesia, Shanghai Children’s Medical Center, Shanghai, China; INIA, SPAIN

## Abstract

**Background:**

Measuring fetal drug concentrations is extremely difficult in humans. We conducted a study in pregnant sheep to simultaneously describe maternal and fetal concentrations of propofol, a common intravenous anesthetic agent used in humans. Compared to inhalational anesthesia, propofol supplemented anesthesia lowered the dose of desflurane required to provide adequate uterine relaxation during open fetal surgery. This resulted in better intraoperative fetal cardiac outcome. This study describes maternal and fetal propofol pharmacokinetics (PK) using a chronically instrumented maternal-fetal sheep model.

**Methods:**

Fetal and maternal blood samples were simultaneously collected from eight mid-gestational pregnant ewes during general anesthesia with propofol, remifentanil and desflurane. Nonlinear mixed-effects modeling was performed by using NONMEM software. Total body weight, gestational age and hemodynamic parameters were tested in the covariate analysis. The final model was validated by bootstrapping and visual predictive check.

**Results:**

A total of 160 propofol samples were collected. A 2-compartment maternal PK model with a third fetal compartment appropriately described the data. Mean population parameter estimates for maternal propofol clearance and central volume of distribution were 4.17 L/min and 37.7 L, respectively, in a typical ewe with a median heart rate of 135 beats/min. Increase in maternal heart rate significantly correlated with increase in propofol clearance. The estimated population maternal-fetal inter-compartment clearance was 0.0138 L/min and the volume of distribution of propofol in the fetus was 0.144 L. Fetal propofol clearance was found to be almost negligible compared to maternal clearance and could not be robustly estimated.

**Conclusions:**

For the first time, a maternal-fetal PK model of propofol in pregnant ewes was successfully developed. This study narrows the gap in our knowledge in maternal-fetal PK model in human. Our study confirms that maternal heart rate has an important influence on the pharmacokinetics of propofol during pregnancy. Much lower propofol concentration in the fetus compared to maternal concentrations explain limited placental transfer in in-vivo paired model, and less direct fetal cardiac depression we observed earlier with propofol supplemented inhalational anesthesia compared to higher dose inhalational anesthesia in humans and sheep.

## Introduction

Propofol is the most commonly used intravenous anesthetic agent and has recently been indicated as the preferred supplemental anesthetic drug for open fetal surgery [1–3]. Traditionally, high-dose volatile anesthesia to the human mother has been used for open fetal surgery in order to promote uterine relaxation, to optimize surgical exposure and to minimize the risk of placental abruption. In a recent study we showed that propofol, in combination with remifentanil, lowered the dose of desflurane required to provide adequate uterine relaxation for open fetal surgery [1]. By using lower doses of desflurane, fetal cardiac function during fetal surgery was better preserved compared to that in fetuses under high-dose desflurane anesthesia [1]. In addition, more stable maternal hemodynamic parameters and less fetal acidosis from this propofol supplemented anesthetic technique were demonstrated in a chronically instrumented maternal-fetal sheep model [2].

Propofol pharmacokinetic (PK) properties are well established in pediatric and adult populations, but little is known of propofol disposition in the unborn fetus especially during open fetal surgery. Many *in vitro* studies using human dually perfused cotyledon demonstrated that propofol is highly lipophilic and rapidly diffuses across the placenta [4, 5]; however, the extent of fetal drug exposure during maternal propofol administration is largely unknown. In the third trimester, the fetal exposure of propofol in utero has been studied in parturients undergoing cesarean section. These studies measured the propofol concentration in maternal and umbilical venous samples at the time of delivery, and then reported fetal/maternal propofol ratios ranging from 0.22 to 0.85 [6–8]. These ratios show large variability because the time of drug administration to the delivery of the baby is variable, while maternal concentrations are changing and steady state conditions cannot be assumed. Moreover, due to rapid physiologic changes during delivery, single pairs of fetal and maternal concentrations may not reflect the drug exposure changes during the post dose time course.

The second trimester is the common period for open fetal surgery which offers many advantages for studying propofol fetal concentrations. After fetal surgery, the fetus is returned to the uterus for continuation of pregnancy. As such, there is no rapid change in pregnancy physiology during fetal surgery, in comparison to multiple changes occurring during cesarean section including delivery of the baby, uterine contraction and removal of the placenta. As compared to lower dose requirements of anesthesia for cesarean section, fetal surgery requires higher doses of anesthetic agents to ensure good uterine relaxation to prevent placental separation. Also the duration of fetal exposure to anesthesia during fetal surgery is longer than anesthesia for a cesarean section due to the more elaborate surgery on the fetus.

Since it is not feasible, safe or ethical to simultaneously collect multiple maternal and fetal blood samples with no direct benefit to a highly vulnerable and high risk human fetal population with a very small blood volume, we conducted this pharmacokinetic study using a chronically instrumented maternal-fetal sheep model. This model is the common animal model for fetal surgery and maternal- fetal hemodynamic studies. Our goal was to characterize propofol pharmacokinetics simultaneously in a paired maternal and fetal model and to identify factors predicting fetal propofol plasma concentrations after maternal intravenous bolus and continuous infusion administration in ewes.

## Materials and Methods

After approval from the Committee for Animal Care at Cincinnati Children’s Hospital Medical Center (protocol number: 0D03027), the pharmacokinetic study was conducted in eight singleton pregnant Dorset ewes at 110–125 days of gestation (term 147–150 days). Anesthesia was achieved with propofol, remifentanil and desflurane and all efforts were made to minimize any potential suffering. We selected the mid-gestational period to mimic the usual time to perform open fetal surgery in humans. Pregnant ewes were purchased from breeder and kept in the animal laboratory facility with unlimited access to food and water. The ewes were kept without food overnight prior to the day of surgery, and prior to anesthetic exposure. All animals were euthanized at the end of the experiment by intravenous injection of pentobarbital and potassium chloride. All procedures were carried out under humane care conditions in compliance with the “Principles of Laboratory Animal Care” by the National Society of Medical Research and the “Guide for the Care and Use of Laboratory Animals” by the National Academy of Sciences.

### Instrumentation

The details for instrumentation of this animal model have been previously described [2]. In short, a venous line and an arterial line were placed into the maternal femoral vein and artery under general anesthesia. After midline laparotomy and mini-hysterotomy, the fetal hind limb was exposed. Catheters were placed in the fetal femoral artery and fetal femoral vein. Through the same hysterotomy, the fetus was repositioned and an umbilical flow probe (4–6 s, Transonic Systems Inc., Ithaca, NY) was placed around the common umbilical artery and secured in place for monitoring of umbilical blood flow. Bilateral uterine arteries were identified, and then uterine flow probes (4–6 s, Transonic Systems Inc., Ithaca, NY) were placed and secured on each vessel. All catheters and probes were tunneled to the flank of the maternal ewe, and stored in a pouch sewn to the skin. The maternal abdominal wall was closed. Following the surgery, the animals were extubated, and allowed to recover prior to returning to their holding cage with free access to food and water. The ewes were assessed during the immediate postoperative period and then daily for 3–5 days for postoperative pain and distress, for which buprenorphine was administered as needed. A recovery of at least 4 days was allowed prior to the pharmacokinetics study as part of a maternal-fetal physiologic study.

### Anesthetic Regimen

General anesthesia was induced with propofol 3 mg/kg and succinylcholine 1.5 mg/kg via the maternal femoral venous line, followed by tracheal intubation and mechanical ventilation with 100% oxygen. The animals were anesthetized with intravenous propofol (450 mcg/kg/min) and remifentanil (0.5 mcg/kg/min) for the first 60 minutes, followed by 1.5 MAC of desflurane (10.2% end-tidal concentration), propofol (75 mcg/kg/min) and remifentanil (0.25 mcg/kg/min) for 90 more minutes to simulate conditions during mid-gestation open fetal surgery. The study drugs were administered via the maternal femoral venous line.

### Pharmacokinetics study

Arterial blood samples were drawn from of the ewe (2 ml) and the fetus (1 ml) using time points obtained from a recently published D-optimal design[9]. Samples were drawn at baseline, and then at 5, 15, 25, 60 minutes after the start of the propofol infusion. After changing the infusion rate at 60 minutes, blood samples were drawn from the ewe and the fetus at 75, 100, 110 and 150 minutes. The propofol infusion was stopped at 150 minutes, and the last blood sample was collected at 180 minutes from the ewe and the fetus. Two investigators were involved in drawing blood samples to ensure simultaneous sample collection from the ewe and the fetus. The actual times for sample collections were prospectively recorded.

All blood samples for propofol analysis were collected in pre-labeled screw top polypropylene tubes containing 10 units of heparin per ml of blood. Plasma was immediately separated by centrifugation and transferred into another pre-labeled polypropylene tube. Plasma samples were stored at -70 degrees Celsius until shipping on dry ice for analysis. Maternal and fetal propofol levels were measured with a sensitive liquid chromatography tandem-mass spectrometry assay (iC42 Integrated Solutions in Clinical Research and Development, University of Colorado, Denver, CO). The intra- and inter-assay variability were lower than 10%. The lower limit of quantification for propofol was 0.05 ng/ml.

### Pharmacokinetic analysis

Population pharmacokinetic analysis was performed by non-linear mixed effects modeling using NONMEM (v 7.2.0; ICON, Ellicott City, MD). An integrated workbench consisting of Pirana (v 2.8.0), Perl-speaks-NONMEM (PsN) (v3.5.3) and R package Xpose4 was used for data visualization, modeling implementation and diagnosis. The first-order conditional estimation with interaction method (FOCE-I) in NONMEM was employed for all model development runs. Steps toward model development were as follows: 1) selection of structural model, 2) selection of the error model, 3) covariate analysis, and 4) validation of the model. For structural model selection, the plasma concentrations from the ewes were first used to find the optimal compartmental model. The fetal data were later included in the analyses to allow the modeling of the relationship between maternal and fetal propofol plasma concentrations. Between subjects variability (BSV) in the different pharmacokinetic parameters was estimated using an exponential error model. For instance, variability in CL was estimated using Eq (1):

$${CL}_{i}={CL}_{pop}\;*\;e^{\eta_{i}}$$ Eq (1)

In which CLi represents the CL of the ith individual, CL_pop_ is the population value of CL, η_i_ is the between-subject random effect for individual i. The distribution of η was assumed to follow a normal distribution with a mean of 0 and variance ω^2^. Residual variability was estimated with a combined proportional and additive error model as shown in Eq (2):

$${C_{ij}={\hat{C}}_{ij}*(1+\varepsilon_{pij})+\varepsilon }_{aij}$$ Eq (2)

where C_ij_ is the jth measured concentration in individual i; Ĉ_ij_ is the model predicted jth measured concentration in individual i; ε_pij_ and ε_aij_ are proportional and additive residual random errors, respectively, for individual i and measurement j; Both residual and random errors were assumed to follow a normal distribution with a mean of 0 and variance σp^2^ and σa^2^, respectively.

Demographic information including gestational age and maternal body weight, as well as hemodynamic measurements including blood pressure, heart rate and uterine blood flow were used in the covariate analysis. The effect of the covariates on parameter estimates was modeled using a normalized power model. The distinction of different models was based on the objective function value (OFV). A decrease in OFV of more than 6.64 points was considered to be statistically significant with p < 0.01 based on a Chi Square distribution. Throughout the model development process, graphical assessment was carried out to evaluate the models.

For model validation, prediction corrected- visual prediction check (pc-VPC) was implemented by PsN [10].A total of 1000 replicates were generated using the final model to simulate expected concentrations for both ewes and fetuses. The simulated median and 90% prediction intervals of propofol concentrations were compared to the observed values in ewes and fetuses, respectively. Non-parametric bootstrap analysis with 1000 resampled datasets was also run for model validation. The estimated medians and 95% confidence intervals (CIs) of parameter estimates were compared with the final model estimates.

## Results

**Fig 1:**
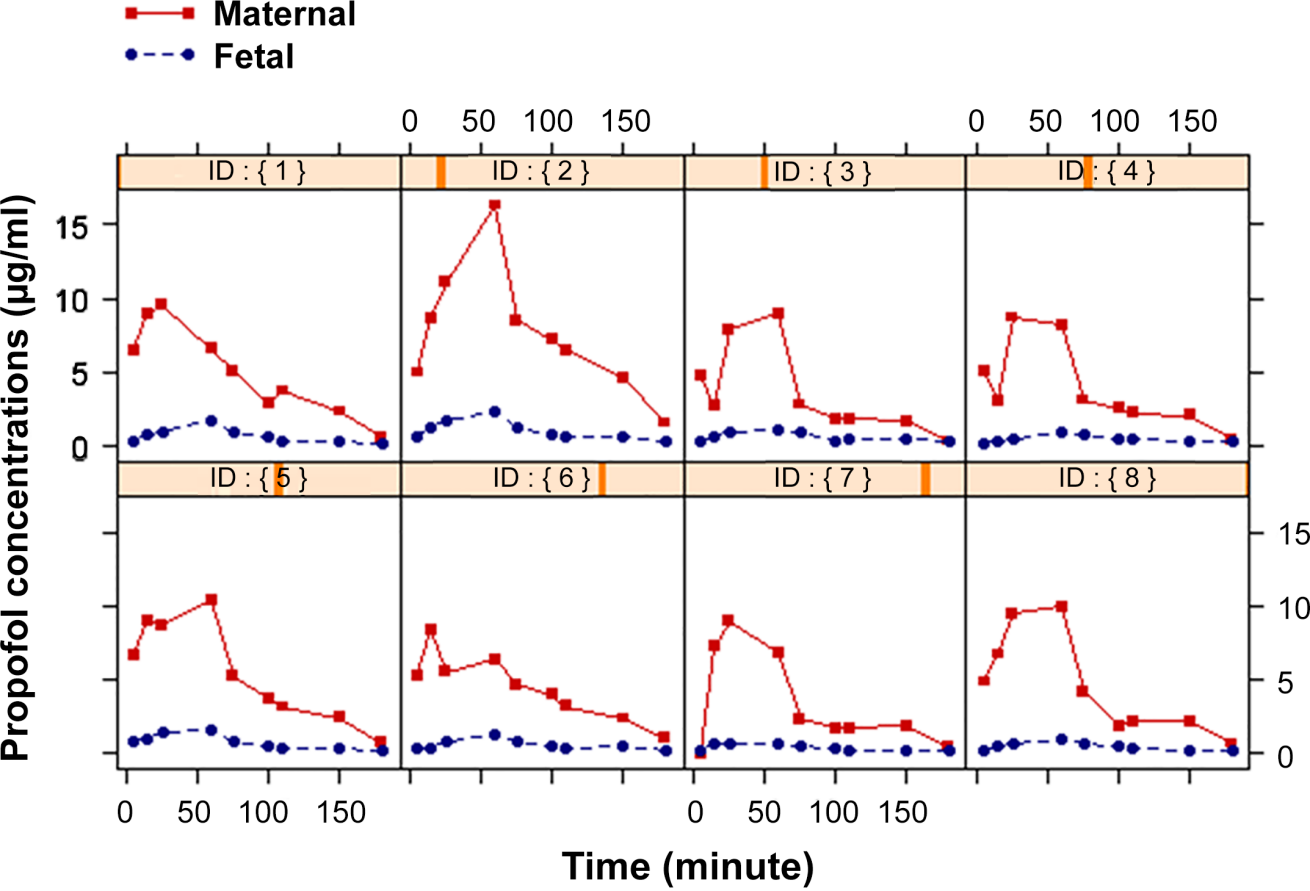
Propofol concentration time profiles for each fetal-maternal sheep unit (n = 8). Propofol was administered to the ewes as a bolus of 3 mg/kg, followed by an infusion of 450 μg/kg/min for 60 minutes. After that, propofol infusion rate was decreased to 75 μg/kg/min for 90 more minutes, and then stopped.

**Fig 2:**
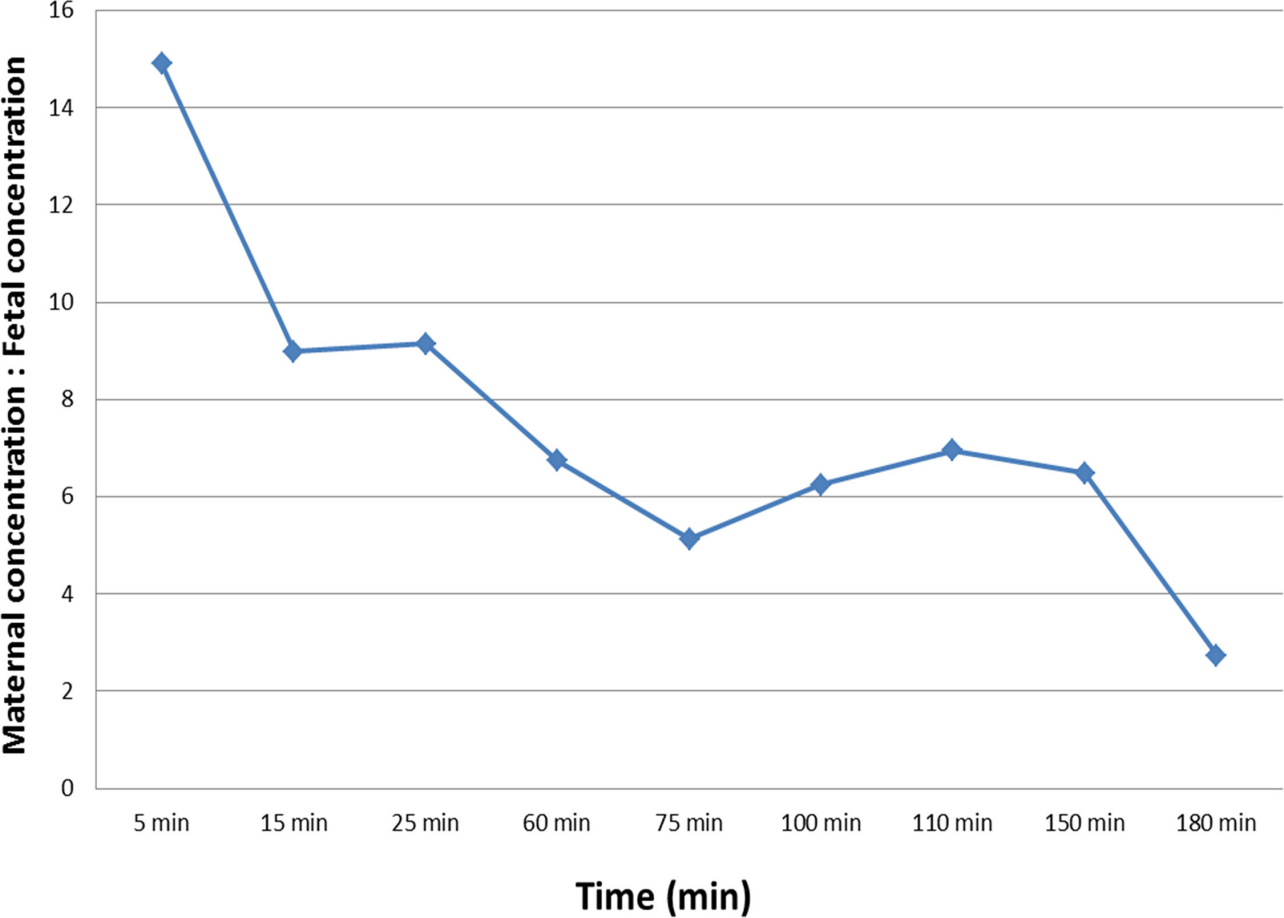
The mean difference between maternal and fetal propofol plasma concentration in sheep after a bolus of propofol 3 mg/kg via the maternal femoral venous line, followed by an intravenous infusion of propofol (450 mcg/kg/min) for 60 minutes and then propofol infusion (75 mcg/kg/min) for 90 more minutes.

A total of 160 propofol concentration measurements (80 pairs of maternal-fetal measurement) were collected from eight mid-gestational ewes and were used for pharmacokinetic analysis. The mean body weight of the pregnant ewes was 71.6 kg (range 60–82 kg, median 72.5 kg) and the mean gestational age was 115.8 days (range 111–118 days, median 116.5 days). Blood samples were collected simultaneously from the maternal and fetal femoral arterial lines. The PK profiles of maternal and fetal propofol from each fetal-maternal unit over time are shown in Fig 1. Propofol plasma concentration in the ewes was 15 times higher than in the fetuses at 5 minutes which was the first sampling time point, and then dropped over time (Fig 2). Table 1 shows mean ± SD of propofol plasma concentrations in the ewes and the fetuses at different time points. The mean of the fetal/maternal propofol concentration ratios during propofol infusion was 0.14 ± 0.06 (range 0.03–0.32). At 30 minutes after the end of the propofol infusion, the mean of the fetal/maternal propofol ratio was 0.37 ± 0.22 (range 0.18–0.81).

**Table 1:** Plasma propofol concentration (μg/ml) in the ewes and the fetuses.

**Time (minutes)**	**Ewes (μg/ml)**	**Fetuses (μg/ml)**	**Fetal: maternal ratio**
**Baseline**	BLQ	BLQ	-
**5**	5.43±2.06	0.35±0.21	0.07±0.04
**15**	6.82±2.57	0.68±0.31	0.11±0.07
**25**	8.71±1.60	0.95±0.41	0.11±0.04
**60**	9.18±3.25	1.31±0.51	0.15±0.06
**75**	4.47±1.95	0.81±0.25	0.20±0.06
**100**	3.33±1.75	0.48±0.12	0.16±0.04
**110**	3.05±1.56	0.40±0.15	0.14±0.06
**150**	2.42±0.93	0.36±0.15	0.15±0.05
**180**	0.70±0.41	0.20±0.06	0.37±0.22

Samples were collected simultaneously from the maternal and fetal femoral arterial lines. Data show as mean ± SD. BLQ = below lower limit of quantification for propofol level (< 0.05 ng/ml).

**Fig 3:**
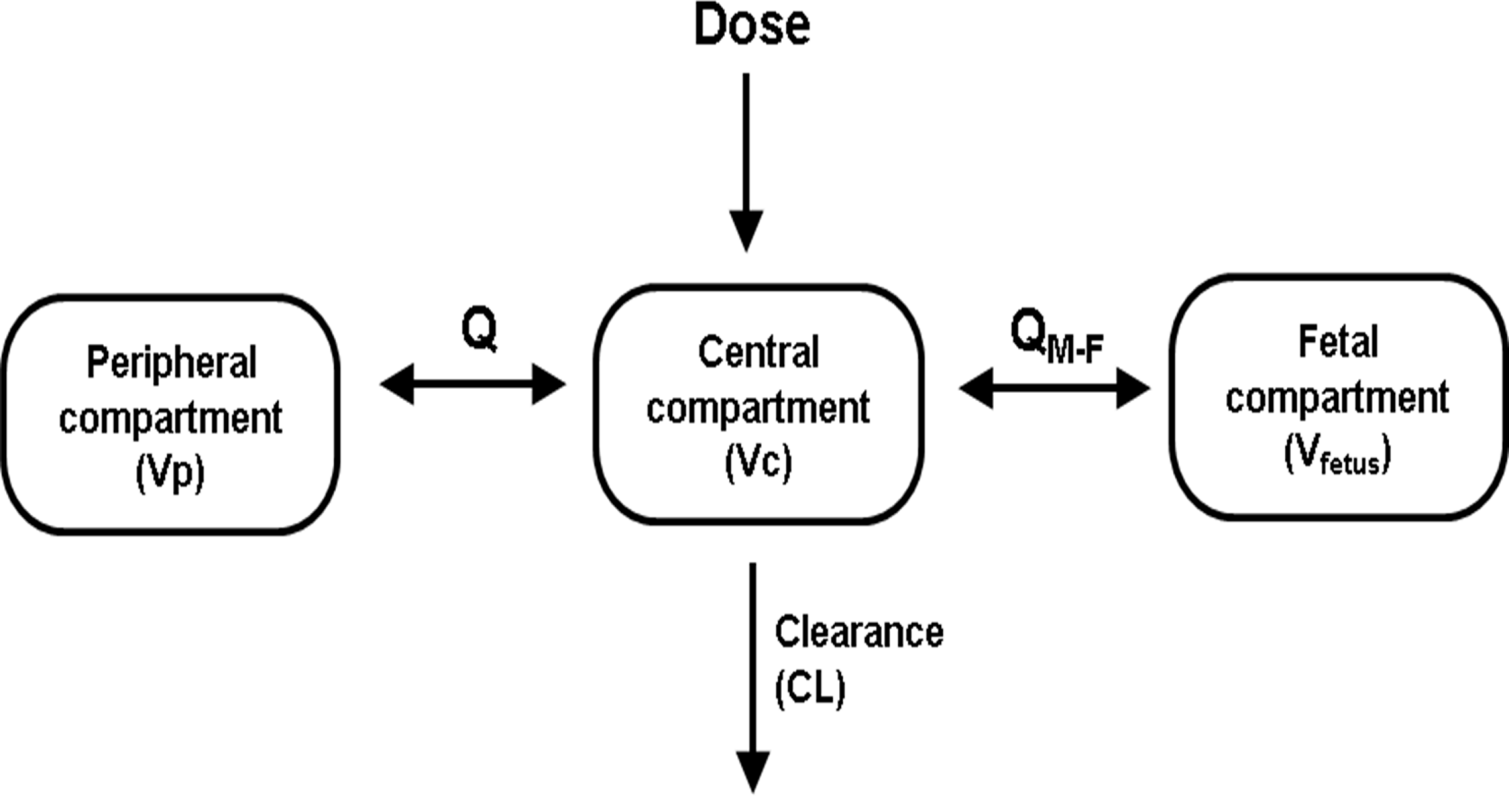
Pharmacokinetic Model. The maternal- fetal pharmacokinetic model of propofol was best fitted using a 2 maternal compartment with a separate fetal compartment model. Vc = maternal central volume of distribution (L), Vp = maternal peripheral volume of distribution (L), Q = inter-compartmental clearance (L/min), CL = clearance from the maternal central compartment (L/min), Q_M-F_ = transfer rate between maternal and fetal compartment (L/min), V_Fetus_ = volume of distribution of fetal compartment (L).

**Fig 4:**
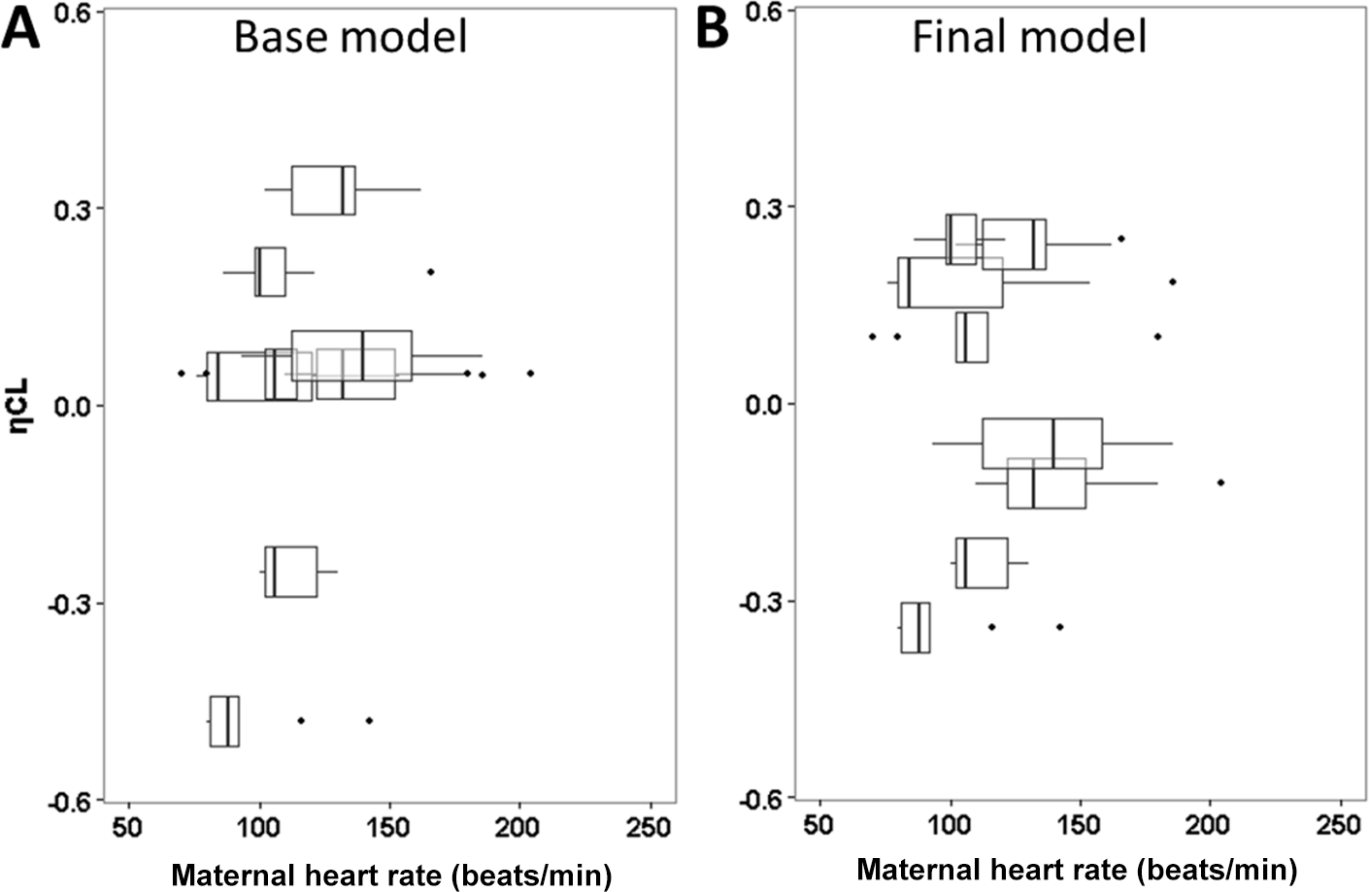
Between-subject random effects (η) for maternal clearance versus heart rate (HR) from the base (A) and final models (B). Each box represents data from one sheep. The lines in the box correspond to median values; the bottom and top of the box are the first and third quartiles (the 25th and 75th percentiles); the upper whiskers extend from the box to the highest value within 1.5 times of inter-quartile range (IQR); the lower whisker extend from the box to the lowest value within 1.5 times of IQR. The individual variability (Random effect, η) for maternal clearance (ηCL) is narrower in the final model than in the base model.

The maternal propofol plasma concentrations were best fitted using a 2-compartment pharmacokinetic model. The model was parameterized in terms of clearance from the central compartment (CL, L/min), central volume of distribution (V_c_, L), peripheral volume of distribution (V_p_, L) and inter-compartmental clearance (Q, L/min) (Fig 3). A 3-compartment model was also tried but did not provide any further improvement. A third fetus compartment was added with parameter Q_M-F_ describing the transfer of propofol between ewe and fetus and parameter V_Fetus_ describing the volume of distribution in the fetus. We also considered including fetal propofol clearance in the model. However, the estimated fetal clearance was very low (< 0.001 L/min) and was with high uncertainty (relative standard error > 100%). The covariate analysis using gestational age, maternal body weight, heart rate, blood pressure and uterine blood flow were separately tested for their influences on the pharmacokinetic parameter estimates. Between-subject random effect (η) for maternal CL is correlated with maternal heart rate with an overall trend of higher η associated with higher heart rate (Fig 4 Panel A). Including maternal heart rate in the final model resulted in lower η (Fig 4 Panel B) and significantly reduced the objective function value (OFV) by 10.3 (P < 0.01). No other covariates were identified to significantly correlate with primary pharmacokinetic parameters. Table 2 summarizes the population parameter estimates for the final model. The typical population values of maternal clearance and the central volume of distribution were 4.17 L/min and 37.7 L, respectively. The typical population values of maternal-fetal clearance (Q_M-F_) and the volume of distribution (V_Fetus_) in the fetus were 0.0138 L/min and 0.144 L, respectively.

**Table 2:** Population pharmacokinetic parameter estimates of propofol in mid gestational maternal-fetal sheep.

Parameter	Final estimate (% RSE)	Bootstrap analysis	
		Median (% Bias)	95% confidence interval
**Fixed effects**			
CL = θ_1_*(HR/158)^θ2^			
θ_1_ (L/min)	4.17 (8.4)	4.14 (0.8)	3.48–5.03
θ_2_	0.764 (28.3)	0.681 (10.8)	0.171–1.211
Vc (L)	37.7 (8.0)	37.6 (0.2)	26.9–44.6
Q (L/min)	1.22 (21.6)	1.23 (1.5)	0.66–2.55
Vp (L)	60.8(21.9)	63.4 (4.3)	40.59–140.54
Q_M-F_ (min)	0.0138 (26.5)	0.0127 (7.6)	0.0082–0.0253
V_Fetus_ (L)	0.144 (6.5)	0.142 (1.3)	0.127–0.164
**Inter-individual variability (% CV)**			
ω1^2^ (CL)	21.8 (17.8)	18.3 (16.1)	4.7–27.6
ω2^2^ (Vc)	0 FIX	-	-
ω3^2^ (Q)	0 FIX	-	-
ω4^2^ (Vp)	0 FIX	-	-
ω5^2^ (Q_M-F_)	66.5 (34.2)	58.7 (11.7)	21.5–96.4
ω6^2^ (V_Fetus_)	0 FIX	-	-
**Residual variability (% CV)**			
σ^2^ (Proportional for ewe)	26.0(26.9)	24.9 (4.3)	18.7–34.2
σ^2^ (Proportional for fetus)	21.8 (32.1)	21.6 (0.6)	14.3–28.7

Abbreviations: CL, maternal clearance; Vc, central volume of distribution; Q, inter-compartmental clearance between central and peripheral compartments; Vp: peripheral volume of distribution; Q_M-F,_ inter-compartment clearance between central maternal compartment and fetal compartment; V_Fetus_, fetal volume of distribution; HR, heart rate; CV: coefficient of variation; RSE, relative standard error.

**Fig 5:**
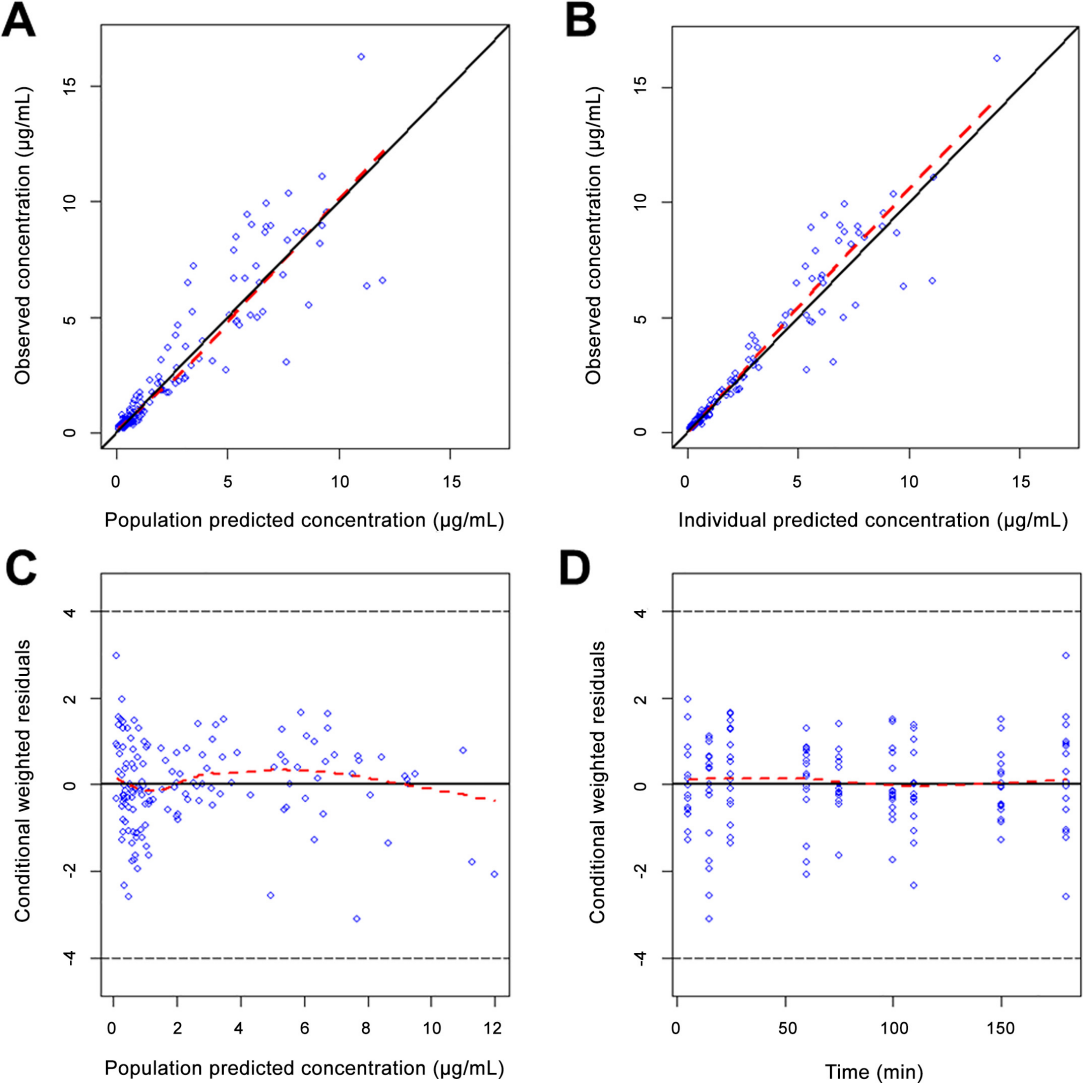
Goodness-of-fit plots for the final PK model. (A) Population prediction versus observed concentration. (B) Individual prediction versus observed concentration. (C) Conditional weighted residuals (CWRES) versus population prediction. (D) Conditional weighted residuals (CWRES) versus time. Dashed red line, a locally weighted least-squares regression; solid black line, line of identity.

**Fig 6:**
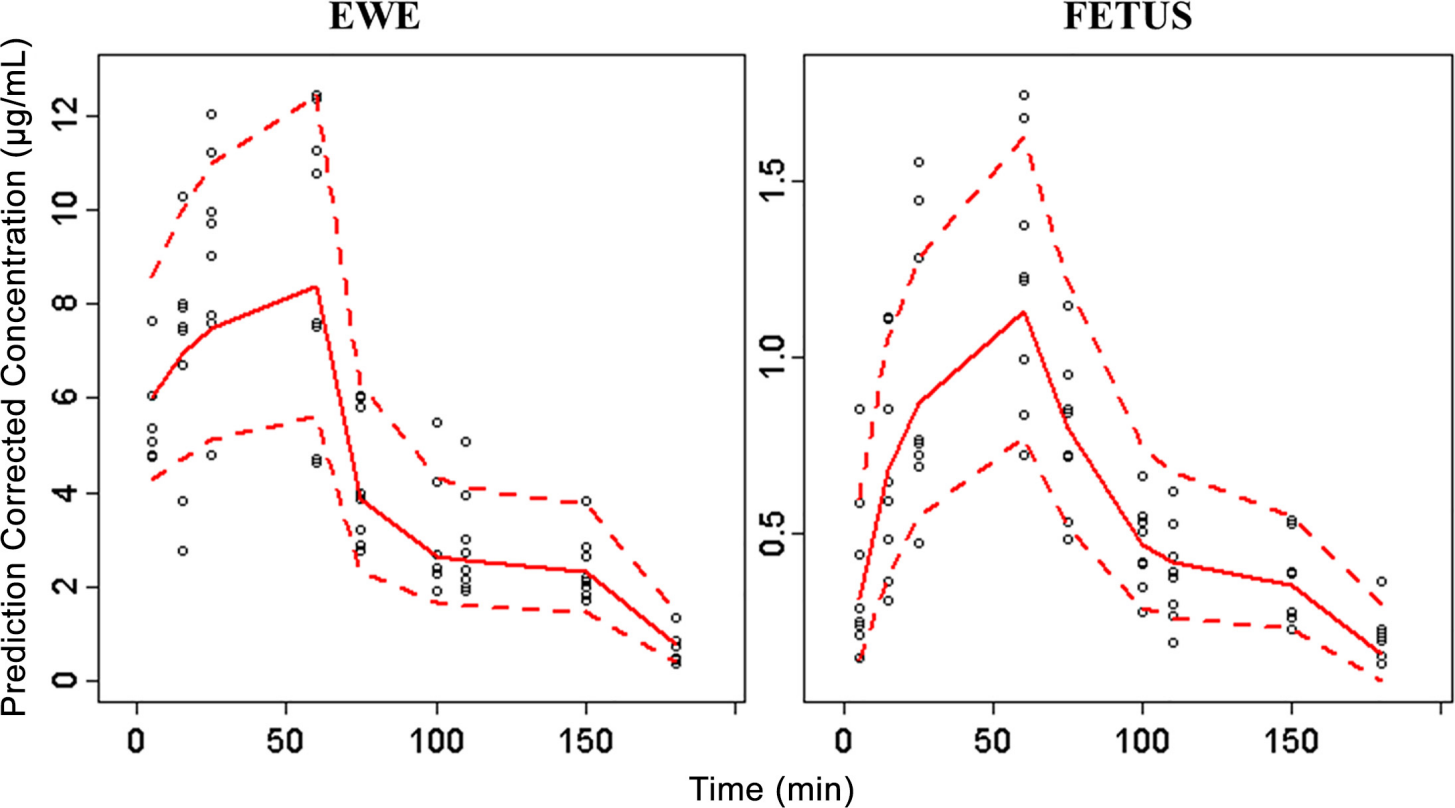
Visual predictive check of prediction-corrected concentration of propofol in ewe and fetus for the final model. Circles demonstrate prediction corrected observations. Red lines demonstrate 5th, 50th and 95th prediction percentiles.

Diagnostic plots indicated no obvious bias for the final model (Fig 5). All the primary PK estimates had less than 10% bias compared to the median from the bootstrap results. Inter-individual variability of maternal clearance differed by 13% to the value in the bootstrap analysis, probably due to the small sample size. A visual predictive plot was further used to evaluate the final model, and to ensure reproducibility. As shown in Fig 6, the 90% prediction intervals generated from the simulations covered the distribution of observed propofol plasma concentrations well in both ewes and fetuses. This indicated that the final model provided a reliable description of the data with good precision of structural model and variance estimates.

## Discussion

### Propofol population pharmacokinetics (PK) in maternal-fetal sheep model

To the best of our knowledge, this is the first study using compartmental analysis to simultaneously describe maternal and fetal propofol PK, and a first attempt to quantitate the effect of covariates on propofol PK during fetal surgery. In this maternal-fetal sheep model, we found that a maternal 2-compartment model with an additional fetal compartment best described the data. Heart rate is a significant predictor of propofol pharmacokinetics in pregnant ewes. This maternal hemodynamic factor might explain underlying hepatic blood flow and clearance of highly lipophilic propofol.

We used a population PK approach for both designing the study and analysis of the data. In the designing phase, we first collected *a priori* PK parameters data from the literature [11–13]. The D-optimal design software, ADAPT (v5, BSR, University of Southern California, Los Angeles), was used to develop dose and optimal sampling times, and to investigate the effects of subject variation and sample size on precision of PK parameter estimates. The details of the optimal design and methodology have been previously described [9]. Our structural 2-compartment model in ewes is consistent with others who reported a two-phase kinetic profile of propofol: a fast distribution phase followed by a slow elimination phase after a single bolus dose, or after stopping a propofol infusion in pregnant ewes [14], non-pregnant sheep [15] and humans[16]. The relationship between the fetal propofol concentration and maternal propofol concentration was successfully described by linking the fetal compartment to the maternal central compartment. Fetal propofol clearance was not included in the final model as we found that the estimation of the fetal clearance was negligible (less than 0.001 L/min), and was with high uncertainty. Propofol is primarily metabolized through glucuronidation with minor contribution from multiple cytochrome P450 enzymes [17, 18]. It has been reported that glucuronidation capacity is very low during fetal development but increases rapidly after birth [19]. In a human PK study in preterm and term neonates, propofol clearance was found to have a very fast maturation trajectory after birth with a clearance of 13.6 ml/kg/min in neonates as compared to 43 ml/kg/min in toddler [20].

In our study, propofol clearance was 4.17 L/min (58.2 ml//min/kg) for a typical ewe with a median heart rate of 135 beat/min and a mean body weight of 71.6 kg. This value is lower than previously reported for clearance in non-pregnant sheep (85.4 ml/kg/min or 2.1 L/min for a 25 kg sheep) [21], and is different from reports of increased propofol clearance during pregnancy in humans [16, 22, 23]. Most of these human studies were conducted in patients undergoing cesarean section. Not only cardiac output and hepatic blood flow increase with pregnancy, blood loss and delivery of the fetus and placenta at cesarean delivery may also contribute to increased clearance. We conducted our study in mid-gestational maternal fetal sheep model in order to mimic the typical time period in which fetal surgery occurs. In our study there was no delivery of the fetus and placenta. However, the presence of the gravid uterus in a supine position during the study could compress venous return, which may have resulted in lower cardiac output and lower hepatic blood flow. Propofol is a high extraction drug where its clearance mainly depends on hepatic blood flow [19]. The lower hepatic blood flow may be one of the reasons for the lower maternal clearance in our model. In addition, as compared to the study by Correia et al. [21] where propofol was used as a sole study agent in non-pregnant sheep, we administered propofol together with remifentanil and desflurane in order to achieve anesthetic condition favorable for fetal surgery. Combination and simultaneous administration of propofol and remifentanil allow synergistic and beneficial anesthesia effects. In healthy volunteers, alfentanil reduced clearance of propofol which caused high plasma propofol concentrations [24, 25]. It is possible that remifentanil also inhibits propofol clearance. In our study, we found that the total volume of distribution was 98.5 L or 1.38 L/kg. This value is higher than the steady state volume of distribution of 1.04 L/kg reported in non-pregnant sheep by Correia et al [21]. Physiologic changes during pregnancy including increased plasma volume as well as the presence of placental tissue likely contribute to the increase in volume of distribution.

Because of its highly lipophilic property and its small molecule weight, propofol diffuses through the placenta rapidly. Andaluz et al. demonstrated that propofol reached the fetus as early as 2 minutes after drug administration [14]. In our study, at the earliest sampling time which was 5 minutes, the mean fetal propofol plasma concentration was 0.35 μg/ml while the mean maternal propofol plasma concentration was 5.4 μg/ml. This fetal propofol concentration was 15 times lower than the maternal propofol concentration. However, 50% of propofol binds to red blood cells while 48% binds with plasma proteins while the remainder represents the free fraction [26]. Since in sheep, fetal hematocrit is much higher than maternal hematocrit [27], the difference in fetal and maternal propofol concentration in whole blood will be less than measuring plasma propofol concentration.

In our model, we found that heart rate significantly predicts the maternal clearance and adding this covariate significantly resulted in model improvement. Propofol is mainly metabolized in the liver, with a high hepatic extraction ratio. As such, increase in hepatic blood flow results in an increase of propofol clearance [3]. A previous study reported that increases in cardiac output and heart rate resulted in increased hepatic blood flow which subsequently led to increased propofol clearance and lower in propofol concentrations [25]. We found no influence of gestational age on fetal propofol levels. All animals in our study were in mid-gestation and there were only small differences in gestational age as well as uterine blood flow. The narrow age range, in combination with small sample size, may have resulted in no significant association detected in the final model.

### Knowledge gap: Fetal/maternal ratio and fetal drug exposure

To date, several studies have made an attempt to identify fetal propofol exposure in humans by using blood collected from the umbilical artery or umbilical vein at the time of delivery. These values were then compared to the maternal propofol level measured at the same time point, and subsequently expressed as the umbilical vein to maternal vein (UV/MV) ratio. There are no human data on propofol pharmacokinetics and fetal drug exposure during mid-gestational pregnancy because of the limitation in methodology and ethical constraints. During mid-gestation open fetal surgery, unborn fetuses are exposed to higher doses of anesthesia (to facilitate uterine relaxation to avoid placental separation with surgical manipulation) for a longer duration than during a typical caesarian section. Though supplementation with propofol reduces need for higher doses of inhalational anesthesia during open fetal anesthesia, there is a critical knowledge gap in our understanding of propofol’s pharmacokinetics in a paired maternal-sheep model. This knowledge will help with better titration of propofol to provide better maternal and fetal hemodynamic stability and preferred effects of anesthesia.

In contrast with fetal anesthesia during mid-gestational fetal surgery; during cesarean section, in order to minimize fetal drug exposure and to keep uterine tone, lower dosages of anesthetic drugs are administered or may be discontinued altogether before delivery. The incision time to delivery time is also short. Therefore the UV/MV ratio, obtained after cesarean section in many studies in humans, is not considered the best indicator as it may not be reflective of the fetal propofol concentration time course observed during prolonged fetal surgery procedures. As maternal and fetal physiologies change significantly during pregnancy, the relationship between the various UV/MV ratios and fetal drug exposure at term cannot simply be extrapolated to other stages of pregnancy. Moreover, the UV/MV ratio is calculated from mean propofol concentrations in fetuses in comparison to mean propofol concentrations in mothers, which is difficult to interpret because serial samples were not taken from the same mother and her fetus.

The chronically instrumented fetal maternal sheep model allowed us to administer anesthetic agents for a long period of time while collecting multiple fetal/maternal blood samples. We selected to instrument pregnant ewes during mid-gestation in order to mimic mid-gestational open fetal surgery. As opposed to cesarean section, anesthetic requirements during open fetal surgery are higher in order to provide uterine relaxation and fetal anesthesia. Due to complexity of the open fetal procedure, the anesthesia administration time is also longer than for cesarean section. During fetal surgery, supplemental propofol lowered the dose of desflurane while provided adequate maternal anesthesia and uterine relaxation without direct fetal cardio-depressive effects of high dose of inhalational anesthesia (1–2). Our finding of much lower propofol concentration in the fetuses as compared to the ewes could partly explain less fetal cardiac depression, and possibly less exposure of anesthesia on the fetal brain.

Given the limited pharmacokinetic data of anesthetic agents in pregnant women, most of our knowledge is derived from pregnant animal models. Species differences are an important consideration when extrapolating data from animal models to the human situation. There are many commonalities including nutrient transport and metabolic function between human placenta and sheep placenta [28]. However, the placental morphology of these two species is very different and there are many unknown mechanisms of drug transport and protein binding. Compared to humans, propofol clearance in sheep is higher [12, 29]. The fetal sheep propofol concentrations in our study were lower than observed in human fetus, despite the fact that we used higher doses. Nevertheless, this animal model allowed us to collect multiple fetal blood samples, and then develop a maternal-fetal compartment PK model.

## Conclusions

For the first time, a paired maternal-fetal PK model of propofol was successfully developed in pregnant sheep. In mid-gestational pregnant ewes, pharmacokinetics of propofol was best described by a two maternal compartments plus one fetal compartment model. Our study demonstrates that maternal heart rate significantly influence pharmacokinetics of propofol during pregnancy by possibly influencing liver blood flow. Much lower propofol concentration in the fetus compared to maternal concentrations explains limited placental transfer in *in-vivo* paired model. Our study narrows the current gap in knowledge of maternal-fetal PK model in human, and could be used to guide titration of propofol during open fetal surgery to provide better and fetal maternal hemodynamics. The propofol concentration in the fetus was much lower than propofol concentration in the ewe. This lower fetal concentration and possibly negligible cardio-depressive effects of propofol are beneficial to fetus during open fetal surgery to avoid higher doses of inhalational anesthesia and associated direct fetal cardiac dysfunction.
